# Variation in parental investment preferences for nestlings of the Gray‐backed Shrike (*Lanius tephronotus*) in alpine environments

**DOI:** 10.1002/ece3.70267

**Published:** 2024-09-18

**Authors:** Jinyuan Zeng, Yueqi Li, Yuhan Gong, Yurou Shi, Hongyuan Feng, Sen Song

**Affiliations:** ^1^ School of Life Sciences Lanzhou University Lanzhou Gansu Province China; ^2^ Gansu Qilian Mountain National Nature Reserve Management Center Zhangye City Gansu Province China

**Keywords:** egg‐laying strategy, environmental factors, feeding preference, fitness, nestling‐rearing strategy

## Abstract

In the northeastern edge of the Qinghai‐Tibet Plateau, the Gray‐backed Shrike, a shrubland bird species of the plateau, confronts harsh living conditions. The impact of such an environment on their reproductive strategies has long intrigued us. This study reveals significant environmental effects on the investment of the Gray‐backed Shrike during their nestling‐rearing and egg‐laying stages. (1) Based on measurements of 215 shrike eggs from 2017 to 2021, we found that under the cold alpine climate, Gray‐backed Shrikes opt for a strategy of larger clutches and bigger eggs as average rainfall decreases. Concurrently, parents display a decreasing hatching order strategy, resulting in significant weight differences among newly hatched nestlings. (2) Marginal and core offspring exhibited no significant differences in fledging conditions. Core offspring generally have a slightly larger physique than marginal ones. However, marginal offspring exhibit the highest growth rate, with similar survival rates across different offspring categories. Parental rearing adopts a nest survival strategy. (3) The food provisioning rate by parents correlates strongly with the number of nestlings, the age of the nestling, and the nest's sex ratio. Differences exist between female and male provisioning rates based on begging intensity and average temperature; higher average temperatures lead to more food, with males providing more food. (4) Factors like nest sex ratio, offspring category, nestling age, and nestling sex influence the feeding preferences of parents. When overall nestling ratios skew towards either male or female, parental feeding preferences align with the actual nest sex ratio. Male and female parental feeding preferences differ based on average temperature and nestling sex. Males consistently exhibit a stronger preference for feeding male nestlings, regardless of the nest's sex ratio. In contrast, females don't show a clear preference, leading to differences in survival rates for different nestling sex under male feeding preferences.

## Graphical abstract

This manuscript studied the breeding behavior in the Gray‐backed Shrike (*Lanius tephronotus*); the research object is valuable. Then, the research conclusion about parental investment preferences has certain reference value.

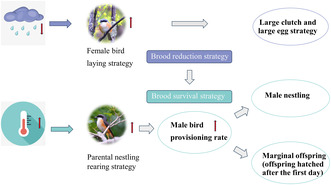

## 1 INTRODUCTION

In the long‐term life history of birds, parents flexibly adjust their life history strategies to adapt to different environmental conditions and ages, balancing between survival and reproduction. Environmental factors impact bird reproduction throughout the breeding period, playing a crucial role in egg‐laying, incubation, and chick‐rearing stages (Nilsson & Svensson, 1996a; Slagsvold, 1986; Slagsvold & Amundsen, 1992; Stenning, 1996). Environmental variations can significantly affect bird reproduction, directly impacting the parents' condition and indirectly influencing reproductive strategies by affecting food resources and other related biological factors (Stearns, 1992). Extreme temperatures, whether too high or too low, present challenges for avian reproduction. In colder conditions, birds dissipate energy faster and need more sustenance to maintain their energy metabolism, while insects are less available (Nilsson & Svensson, 1996a; Turnock et al., 1983). In contrast, in hotter conditions, parents struggle to cool down. Additionally, they spend more time shading their nestlings, reducing the time they can allocate to find food, which adversely affects nestling growth (Stevenson & Bryant, 2000). Limited rainfall doesn't favor vegetation growth, impacting the birds' food supply, and indirectly affects their breeding timing and success rate (Zuckerberg et al., 2018). Prolonged and heavy rainfall raises the humidity in the air, increasing the risk of diseases for nestlings and making it harder for parents to find and deliver food (Schöll & Hille, 2020). High wind speeds hinder nestling insulation; in some species, parents are adapted to nest in locations shielded from strong winds (Heenan & Seymour, 2012; Saraux et al., 2016).

Throughout the extended life history of birds, parent birds adaptively adjust their egg‐laying strategies in response to varying environmental conditions and age, striking a balance between survival and reproductive success. Bird egg‐laying strategies primarily fall into two categories: “many small eggs” and “few large eggs” (Slagsvold & Amundsen, 1992). While there is considerable variability in the number of eggs per clutch among different bird species, the number of eggs laid by most bird species is relatively consistent (Hill, 1984). Environmental temperature influences the clutch size; birds breeding in hotter regions tend to lay fewer eggs, as the ambient temperature surpasses the eggs’ physiological threshold. In contrast, cooler temperatures can maintain the eggs in a highly viable pre‐hatch state, allowing birds in cooler areas to lay more eggs (Cooper et al., 2005). This principle is well‐illustrated by studies on the Black Redstart (*Phoenicurus ochruros*) (Song et al., 2016).

Birds’ incubation strategies can be categorized into synchronous and asynchronous incubation (Slagsvold, 1986; Trivers, 1974), with each approach yielding different outcomes (Slagsvold & Amundsen, 1992). Altricial bird species tend to adopt asynchronous incubation strategies, such as the Zebra Finch (*Taeniopygia guttata*) found in the Passeriformes order (Gilby et al., 2011; Slagsvold et al., 1992). This gives rise to a nestling hierarchy where nestlings hatched on the first day are termed “core offspring,” and those hatched subsequently are called “marginal offspring.” Establishing this hierarchy helps reduce competition and energy expenditure among siblings, enhancing offspring survival (Podlas & Richner, 2013).

Birds adjust their nestling‐rearing strategies in various ecological environments to enhance their fitness. Studies indicate that parent birds might modify the hatching timing or the order of egg weight to implement either nest survivorship or nest reduction strategies (Stenning, 1996). In favorable environments, birds are more likely to adopt a nest survivorship strategy, evenly distributing resources or favoring weaker nestlings. With abundant food, parents control their brooding time and adjust the hatching rate to minimize asynchronous hatching, favoring synchronous nestling‐rearing. This increases the likelihood of successfully feeding all nestlings, a hypothesis validated in studies on the Common Kestrel (*Falco tinnunculus*; Martinez‐Padilla & Vinuela, 2011). However, in less favorable conditions, they tend to use a nest reduction strategy, channeling resources to higher‐quality offspring to gain the largest benefits (Clark & Wilson, 1981). In food‐scarce situations, parents prioritize feeding core offspring and stronger marginal offspring, resulting in the highest mortality rate among the weakest marginal nestlings, a strategy observed in the Horned Lark (*Eremophila alpestris*; Du et al., 2014).

In our study, we delved into how environmental factors shape the overall nestling‐rearing strategies of parent birds in extreme environments. Additionally, we conducted an analysis of feeding preferences to reveal any differences in fitness between male and female parent birds. This study enhances understanding of the intricate relationship between sex and the environment within ecosystems, contributing to the broader knowledge of reproductive ecology in high‐altitude birds.

## 2 MATERIALS AND METHODS

### 2.1 Study area

The study site is located in Luqu County, Hezuo City, Gannan Tibetan Autonomous Prefecture, Gansu Province, China (101°35′–102°58′ E, 33°58′–34°48′ N) with an average altitude of 3500 m. Luqu County comprises basins and mountainous terrains, with a high west and a lower east. The western region consists of plateau mountains, while the eastern part lies in the Taohe River basin (Hu et al., 2022). Positioned in the northeastern part of the Qinghai‐Tibet Plateau, the county features a plateau climate typical of alpine meadows, characterized by long winters, short summers, and considerable daily temperature fluctuations. The annual average temperature stands at 2.3°C, with yearly precipitation ranging between 633 and 782 mm (Ren et al., 2016).

### 2.2 Study species

The Gray‐backed Shrike (*Lanius tephronotus*) is a carnivorous bird species within the order Passeriformes and family Laniidae (Del Hoyo et al., 1992). Typically, the Gray‐backed Shrike breeds between May and July, constructing nests on shrubs such as the Sea Buckthorn (*Hippophae rhamnoides*) and Japanese Barberry (*Berberis thunbergii*). During the breeding season, their primary nourishment for nestlings consists of locusts (*Locusta migratoria*), wasps (*Vespoidea*), and caterpillars (*Lepidoptera*; Hu et al., 2022; Lu et al., 2010).

### 2.3 Field data collection

During the breeding season of the Gray‐backed Shrike from May to July in 2017–2021, nests were actively searched within the study area, and their locations were recorded using the Global Positioning System (GPS). Regular nest visits commenced from the onset of egg‐laying by the parent birds, with measurements conducted every other day during the incubation period and daily visits during the nestling period. The number and identification of eggs in each nest were documented. Using calipers, the length and width of the eggs were measured to the nearest 0.01 cm, and initial egg weights were determined using an electronic scale to the nearest 0.1 g. Once the nestlings hatched, we used non‐toxic, colored markers to identify them. Different numbers were marked on the tops of their heads, and varying numbers of lines were drawn on their tarsi, and growth metrics were recorded. Blood samples were taken from the nestlings around the age of 10 days to minimize the impact of sampling on nestling growth (Data S1).

### 2.4 Parental feeding data

Upon locating a nest, ZX1 Digital camcorders (Eastman Kodak Company) was set up to record the nest activities throughout the day. Playback of these recordings allowed the extraction of data including the frequency of parental feedings, the quantity of food provided in each feeding session, and the identity of the nestling receiving the food. This information was used for further analysis of variations in parental investment and feeding preferences.

### 2.5 Environmental data

**FIGURE 1:**
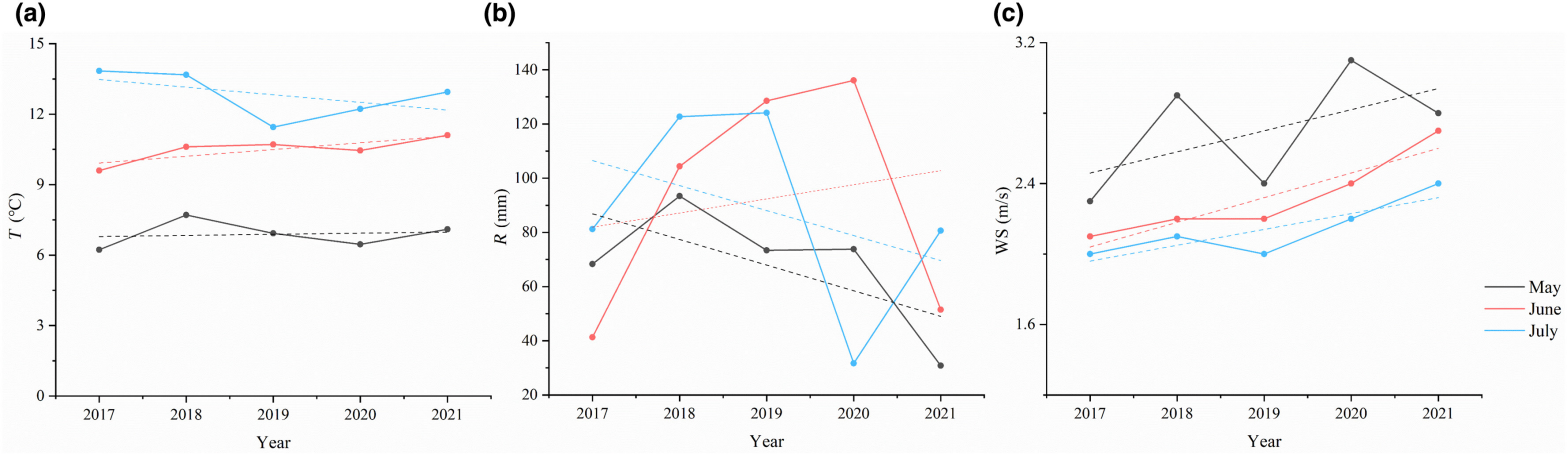
Changes in reproductive season environmental factors.

Climate data for Lüqu County were sourced from the Geographical State Monitoring Cloud Platform (http://www.dsac.cn/). The temporal scale of environmental factors was analyzed on a monthly basis, covering the breeding season of the Gray‐backed Shrike from May to July. Spatially, the environmental factor data were collected within a 20‐km radius centered on the study location. A multicollinearity analysis was performed to eliminate variables with high collinearity (|*r*| > .8), retaining average temperature, average rainfall, and average wind speed as the variables of interest. Figure 1 shows that these climate variables over the months of May, June, and July for 5 years revealed fluctuations in the temperature during May, an overall increasing trend in June, and a general decrease in July. Average rainfall showed a decline in May, an overall increase in June, and a decline in July. Average wind speed increased consistently over the 3 months. Significant differences were observed between the months in terms of temperature and average wind speed (*p* < .01; Figure 1).

### 2.6 Data analysis

#### 2.6.1 Bird blood DNA extraction

DNA is extracted from the blood of the Gray‐backed Shrike, followed by PCR amplification, electrophoresis, and silver staining to determine the sex of the nestlings and adult birds. The extraction process is carried out according to the instructions of the Tiangen Blood DNA Extraction Kit (DP201101X).

Statistical analyses were performed using SPSS Statistics 26 and R 4.2.2. Data were presented as “mean ± SD”. Prior to statistical analysis, homogeneity of variance was ensured. The level of statistical significance was set at *α* = .05, with all tests being two‐tailed.

Calculation of Egg Volume and Initial Weight: We tracked nests with known egg‐laying start dates daily, marking the sequence number of each egg with a marker and measuring the egg's length and width using a vernier caliper. The egg volume (*V*) was calculated using the formula *V* = Kv × *L* × *B* ^2^ (cm^3^), where Kv is the egg volume constant, taken as 0.510 (Hoyt, 1979), *L* is the egg length, and *B* is the egg width. The initial egg weight (*W*) was calculated using the formula *W* = Kw × *L* × *B* ^2^ (g), where Kw = Kv initial/Kw initial. For the Gray‐backed Shrike, Kw was calculated to be 0.529 (Barth, 1953).

The growth curve of nestlings was modeled using the logistic growth curve: *W* = *K*/(1 + exp (*a* – *t* * *d*)), where *K* represents the largest value a nestling's particular metric can attain, a represents the time when the nestling's growth rate is at its peak, *t* represents the rate of nestling growth, and *d* stands for the nestling's age in days. To investigate the influence of hatching order and the number of nestlings in a nest on nestling development, we examined growth curve parameters across different hatching sequences and brood sizes.

A one‐way analysis of variance (ANOVA) was used to test for significant differences in the number of eggs per nest, and egg volume across different years, to analyze changes in parental egg‐laying strategies, and differences in nestling weight, wing length, and beak gape. To determine the primary environmental influencing factors, we conducted a multicollinearity analysis on the following environmental variables: monthly average daylight hours, monthly average humidity, highest monthly average rainfall, lowest monthly average rainfall, monthly average rainfall, highest monthly average temperature, lowest monthly average temperature, monthly average temperature, highest monthly average wind speed, lowest monthly average wind speed, and monthly average wind speed. We excluded factors with high collinearity, such as daylight hours and humidity, and selected variables with low collinearity (|*r*| < .8, VIF < 10). The final three main environmental factors retained were monthly average rainfall, monthly average temperature, and monthly average wind speed.

We adopted a Generalized Linear Mixed Model (GLMM) approach (Nakagawa & Schielzeth, 2013) to analyze factors influencing the Gray‐backed Shrike's nestling‐rearing process and the strategies adopted by the parents. Nests were categorized based on sex ratios into female‐biased and male‐biased nests. A GLMM analysis was performed on the parental feeding preferences to investigate whether there is a sex preference in the offspring they feed. We conducted model analyses on clutch size to investigate their main influencing factors. Factors such as nest initiation time, clutch size, egg volume, average temperature, average rainfall, and average wind speed were selected as fixed effects (independent variables). Year, nest location, and nest ID, due to repeated measurements, were set as random effects (dependent variables). The optimized models are presented in the Results section.

We also conducted model analyses on nestling weight, female provisioning preference, male provisioning preference, female feeding rate, and male feeding rate to investigate their main influencing factors. Factors such as brooding start time, nest identification, nest location, brood type, brood sex ratio, nestling sex, feeder identification, parental feeding, estimated food amount, nestling begging intensity (Table S1), number of nestlings raising their heads, nestling age, average temperature, average rainfall, and average wind speed were selected as fixed effects (independent variables). Year, nest location, and nest ID, due to repeated measurements, were set as random effects (dependent variables). The optimized models are presented in the Results section. This approach provides a comprehensive understanding of the factors influencing parental care.

## 3 RESULTS

### 3.1 Differences in egg volume and egg weight across different years

Between 2017 and 2021, a total of 215 shrike eggs were measured. On average, the annual clutch size for the shrike was 4.87 ± 0.67 eggs (*n* = 215), with an average egg volume of 4.3 ± 0.43 cm^3^ (*n* = 215), and the average initial egg weight was 4.49 ± 0.45 g (*n* = 215). Significant differences existed in the clutch size, egg volume, and initial egg weight across the years (*p* < .05; Table 1). The year 2021 recorded the highest average values for all three metrics: clutch size was 5.06 ± 0.75 eggs, egg volume was 4.44 ± 0.51 cm^3^, and the initial egg weight was 4.63 ± 0.54 g (*n* = 69). In contrast, the year 2017 had the lowest values, with an average clutch size of 4.56 ± 0.51 eggs, egg volume of 4.14 ± 0.46 cm^3^, and an initial egg weight of 4.32 ± 0.47 g (*n* = 27). Clutch size, egg weight, and egg volume have all shown an annual increase, indicating that parent birds have adopted a strategy of producing larger clutches with larger eggs. Upon conducting the LSD (Least Significant Difference) pairwise comparison analysis, significant differences were found in the egg volume and initial egg weight among the different years (*p* < .05). The clutch sizes for the years 2017–2019 were significantly different from that of 2021 (*p* < .05; Table 1).

**TABLE 1:** Differences in clutch size, egg volume, and egg weight across year.

Year	2017		2018		2019		2020		2021		*F*	*P*
	M ± SD	*n*	M ± SD	*n*	M ± SD	*n*	M ± SD	*n*	M ± SD	*n*		
CS	4.56 ± 0.51	27	4.79 ± 0.41	38	4.82 ± 0.73	33	4.92 ± 0.68	48	5.06 ± 0.75	69	3.25	.01
EV	4.14 ± 0.46		4.25 ± 0.30		4.25 ± 0.38		4.26 ± 0.38		4.44 ± 0.51		3.25	.01
EW	4.32 ± 0.47		4.44 ± 0.32		4.43 ± 0.39		4.45 ± 0.40		4.63 ± 0.54		3.21	.01

*Note*: *p* < .05 represents a significant difference.

Abbreviations: CS, clutch size; EV, egg volume; EW, egg weight.

### 3.2 Factors influencing clutch size

Average rainfall and nest initiation time had impacts on clutch size. Average wind speed, position in the laying sequence, and nest initiation time exhibited a significant positive correlation with clutch size (*p* < .01). Conversely, average rainfall demonstrated a significant negative correlation with clutch size (*p* < .05; Table 2).

**TABLE 2:** Generalized Linear Mixed Model (GLMM) analysis of factors influencing clutch size.

Fixed effects	*β* ± SE	*t*	*n*	*p*	95%CI
Intercept	4.659 ± 0.120	38.816	215	<.001	4.402 ± 4.943
*R*	−0.152 ± 0.068	−2.253	215	.025	−0.285 ± 0.006
Sequence	0.059 ± 0.021	2.767	215	.006	0.017 ± 0.101
Time	0.661 ± 0.187	3.541	215	<.001	0.290 ± 1.033
*T*	0.034 ± 0.114	0.302	215	.763	−0.192 ± 0.260
WS	0.963 ± 0.295	3.266	215	.001	0.370 ± 1.790

Random effects	*β* ± SD	*n*	VCA (%)
Nest ID	0.435 ± 0.660	215	69.92
Year	0.001 ± 0.001	215	0.01
Residual	0.187 ± 0.433	215	30.07

*Note*: VCA% represents the proportion of total variance explained by each component. 95%CI represents the 95% confidence interval for the estimates.

Abbreviations: *R*, average rainfall; *T*, average temperature; WS, average wind speed.

### 3.3 Nestling logistic growth curve

**FIGURE 2:**
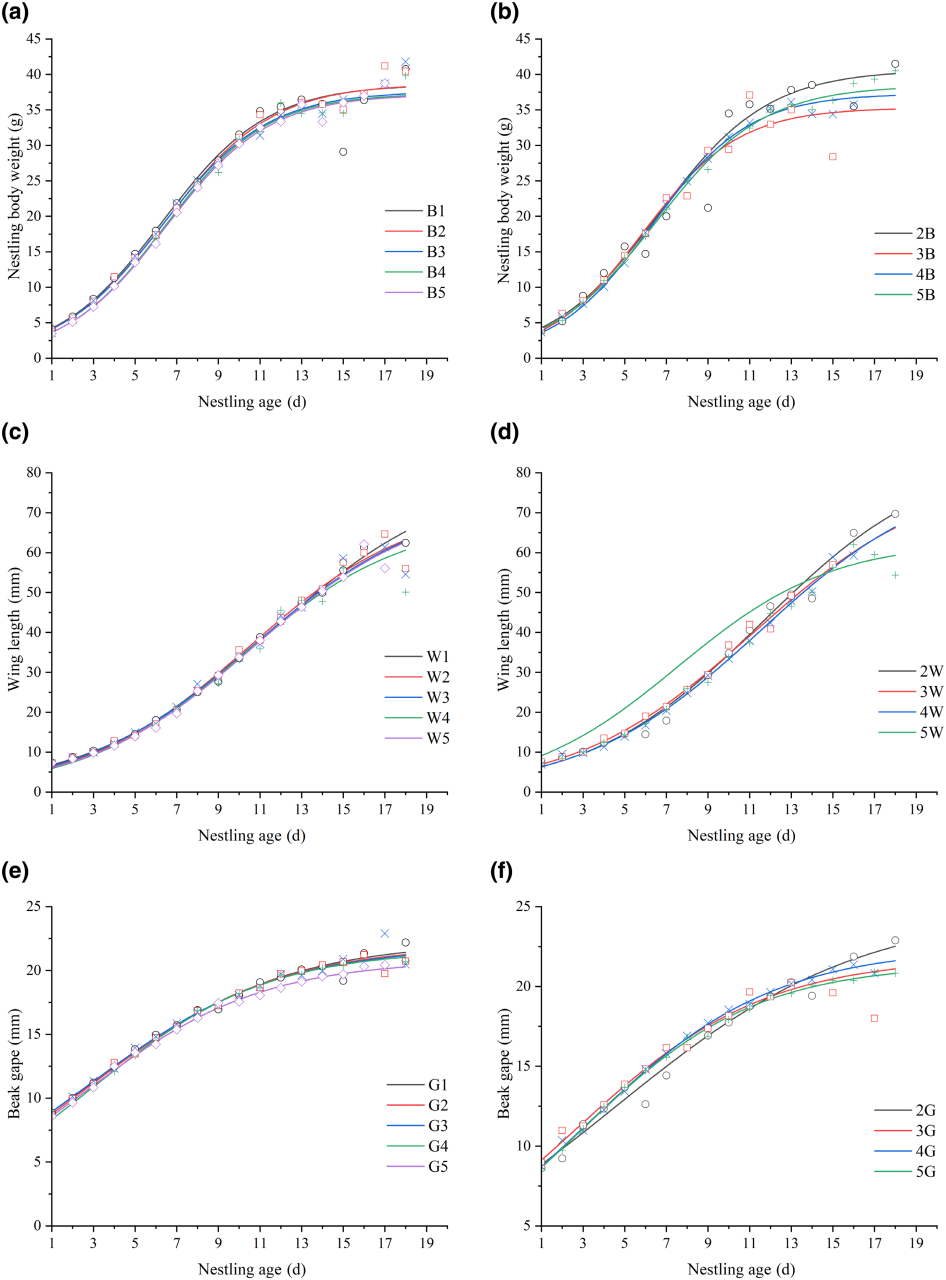
Logistic growth curves for nestlings. (a, b) Weight growth by hatching order and brood size, respectively. (c, d) Wing length growth by hatching order and brood size. (e, f) Beak gape growth by hatching order and brood size. B1–5, W1–5, G1–5: The first five positions in the hatching order for body weight (B), wing length (W), and beak gape (G). 2–5B, 2–5 W, 2–5G: Brood sizes ranging from 2 to 5 eggs for body weight (B), wing length (W), and beak gape (*G*).

Between 2017 and 2021, a total of 63 valid nest data sets were obtained from fieldwork. The logistic equation relating nestling hatching sequence to weight indicates that the first‐hatched nestling had the largest fledging weight, while the weight of the last‐hatched nestling is the lowest, showing a declining trend in weight with hatching order. Analysis of variance on fledging weight revealed no significant differences among the nestlings (Figure 2). In terms of growth rate, the last two hatched nestlings grew at a faster rate. There were no significant weight differences among nestlings from nests with different brood sizes, with nestlings from two‐egg clutches having the largest fledging weight and the slowest growth rate (Figure 2). Significant differences were observed in nestling birth weights, with the first two nestlings weighing more than the subsequent ones (*p* < .05; Figure 2). Male nestlings had higher birth and fledging weights compared to female nestlings, but these differences were not statistically significant (Figure 2).

The logistic equation for nestling hatching sequence against wing length shows that the first‐hatched nestling has the longest wing span at fledging, while the last two nestlings have the shortest. The fifth nestling exhibited the fastest wing growth rate. There were no significant differences in wing length relative to brood size (Table S2).

**FIGURE 3:**
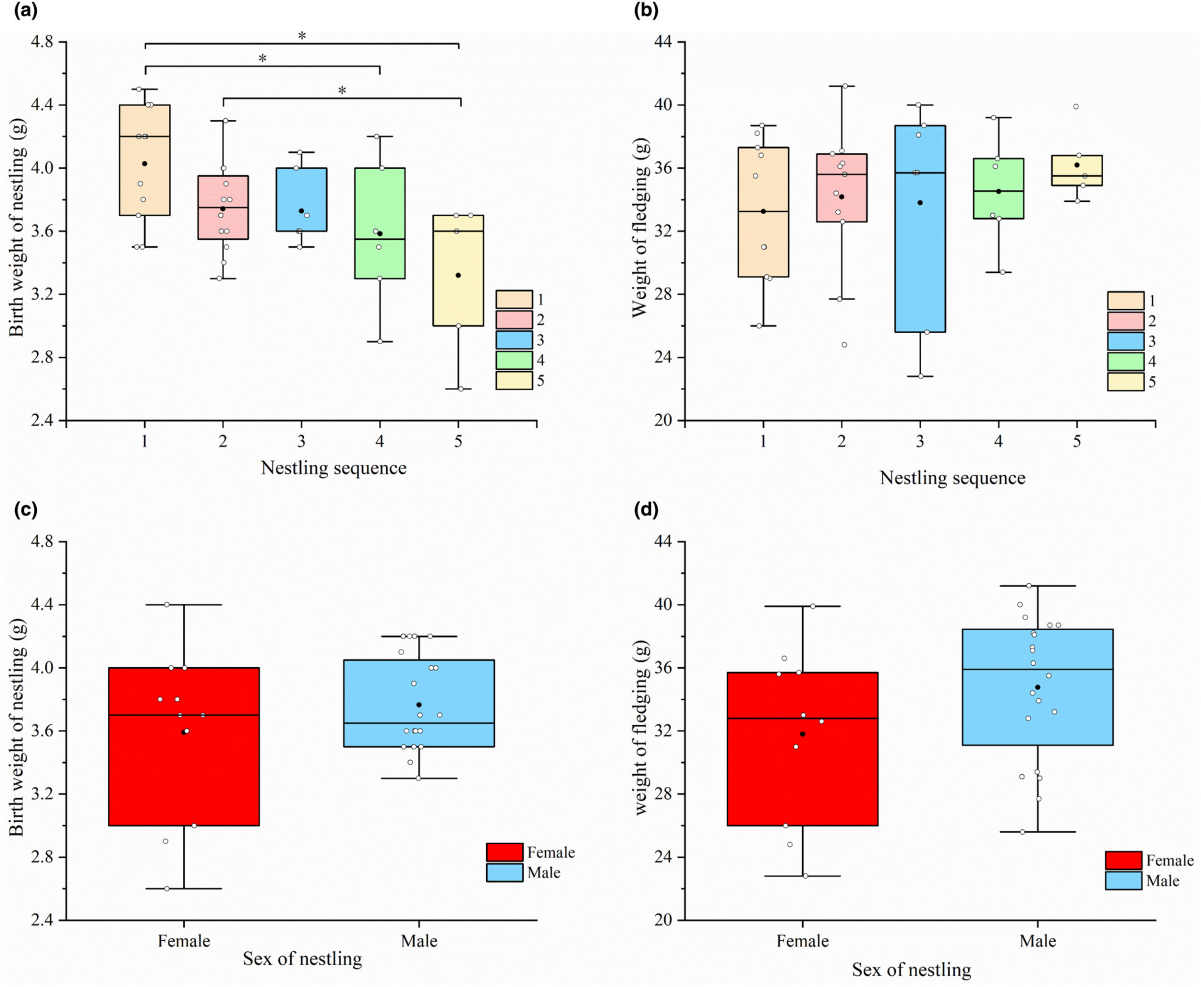
Birth weight and fledging weight of nestlings. (a) Differences in hatchling birth weights; (b) Differences in fledging weights; (c) Differences in birth weights by sex; (d) Differences in fledging weights by sex. 1–5 represent hatchlings with different hatching orders, and * indicates significant differences.

For the relationship between hatching sequence and beak gape based on the logistic equation, the first‐hatched nestling had the largest beak gape, while the last one had the smallest, displaying a trend of decreasing beak gape with increasing hatching order. The fourth and fifth nestlings grew their bills at a faster rate. No significant differences were found among fledglings' beak gapes (Table S2). The relationship between brood size and beak gape followed the same trend as that between hatching sequence and wing length (Figure 3).

### 3.4 Factors affecting nestling weight

Average temperature and nestling age significantly influence nestling weight. There is a notable negative correlation between average temperature and nestling weight (*p* < .05). A significant positive relationship was observed between nestling age and weight (*p* < .01), reflecting the typical growth pattern where nestlings gain weight as they age, provided they receive sufficient food (Table 3).

**TABLE 3:** Generalized Linear Mixed Model (GLMM) analysis of factors influencing nestling weight.

Fixed effects	*β* ± SE	*t*	*n*	*p*	95%CI
Intercept	20.300 ± 0.518	39.223	760	<.001	19.186 ± 21.338
Brood size	−0.368 ± 0.317	−1.159	760	.250	−1.060 ± 0.268
NA	2.666 ± 0.027	98.653	760	<.001	2.613 ± 2.720
*T*	−0.650 ± 0.221	−2.936	760	.004	−1.129 ± −0.210
*R*	0.008 ± 0.006	1.309	760	.193	−0.005 ± 0.022

Random effects	*β* ± SE	*n*	VCA (%)
Nest ID	4.457 ± 2.111	760	33.72
Nestling number	0.391 ± 0.625	760	2.96
Residuals	8.370 ± 2.893	760	63.33

Abbreviations: NA, nestling age; *R*, average rainfall; *T*, average temperature.

### 3.5 Differences in nestling survival rates

There is no overall difference in survival rates between core and marginal nestling offspring. However, annually, the survival rate of core offspring is slightly higher than that of marginal offspring, with 2021 being an exception where the survival rate of marginal offspring surpassed that of core offspring. A difference exists in survival rates between male and female nestlings. Male nestlings exhibit a survival rate higher than female nestlings, exceeding by 27%. Over the span of 5 years, male nestlings generally outperformed the females, with 2020 being an exception when female nestlings had a marginally higher survival rate than their male counterparts (Table 4).

**TABLE 4:** Nestling survival rate.

Year	Core offspring	Marginal offspring	Overall survival rate	Male	Female	Overall survival rate
2018	0.65	0.55	0.61	0.80	0.36	0.57
2019	0.82	0.75	0.80	0.86	0.50	0.75
2020	0.64	0.43	0.59	0.93	1.00	0.95
2021	0.63	0.92	0.71	0.89	0.78	0.83
Sum	0.70	0.73	0.71	0.88	0.61	0.77

### 3.6 Factors influencing parental feeding amount

The data analysis incorporated 13 nest video recordings, accumulating 1414 hours of valid footage and extracting 6829 meaningful behavioral events. The analysis of male and female parental feeding amounts aimed to discern the factors causing differences between them (Table S3). The model indicated that the female feeding amount was influenced by five factors: number of nestlings, age of nestlings, nest sex ratio, average wind speed, and begging intensity. Specifically, the female's feeding amount showed a strong positive correlation with the number of nestlings, age of nestlings, and nest sex ratio (*p* < .01) and was negatively correlated with begging intensity and average wind speed (*p* < .05).

**FIGURE 4:**
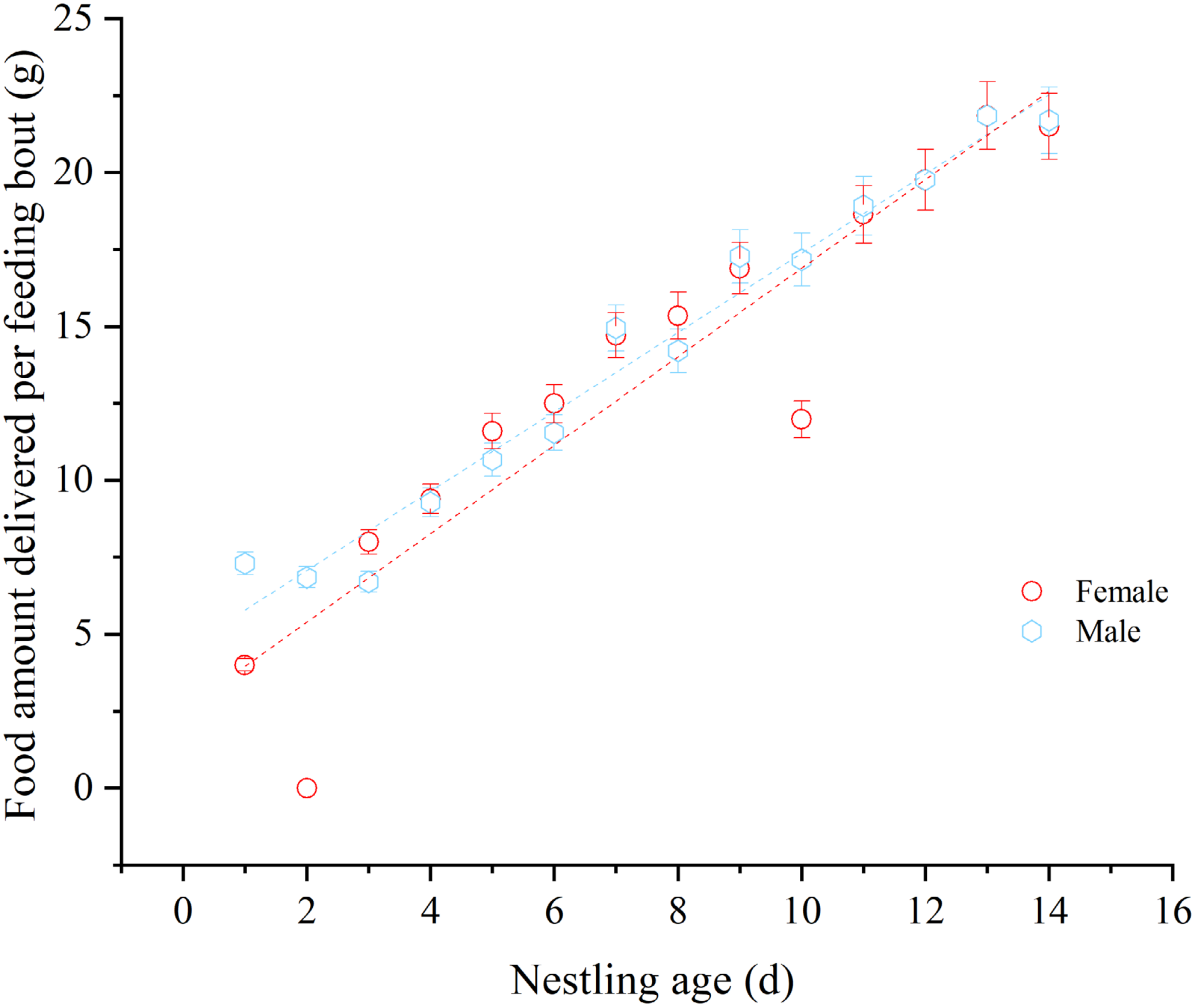
Parental feeding amount.

Additionally, the male feeding amount was influenced by the age of nestlings, number of nestlings, nest sex ratio, average wind speed, and average temperature. As nestling age, sex ratio, and number of nestlings increase, and as the average temperature rises, the male feeding amount also increases (*p* < .01). However, male feeding had a significant negative relationship with the average wind speed (*p* < .05; Table 5). During the early stages of nestling care, males fed more than females, but no significant differences were observed in the later stages (Figure 4).

**TABLE 5:** Factors influencing the amount of food provided by parents.

Female					Male			*p*
Fixed effects	*β* ± SE	*t*	*n*	*p*	*β* ± SE	*t*	*n*	
(Intercept)	17.734 ± 2.616	6.778	1199	<.001	16.724 ± 2.291	7.299	1377	<.001
Begging intensity	−1.283 ± 0.329	−3.903	1199	<.001	0.122 ± 0.101	1.207	1377	.228
NA	0.559 ± 0.126	−4.430	1199	<.001	1.084 ± 0.278	−3.907	1377	<.001
Sex ratio	3.100 ± 0.664	4.666	1199	<.001	2.693 ± 0.639	4.213	1377	<.001
Brood size	3.291 ± 0.883	3.725	1199	<.001	2.092 ± 0.688	3.040	1377	.003
*R*	−0.019 ± 0.049	−0.393	1199	.695	−0.027 ± 0.042	−0.642	1377	.522
*T*	0.936 ± 0.508	1.845	1199	.067	1.387 ± 0.462	3.000	1377	.003
WS	−12.623 ± 5.913	−2.135	1199	.034	−12.038 ± 5.410	−2.225	1377	.027

Random effect	*β* ± SD	*n*	VCA (%)	*β* ± SD	*n*	VCA (%)
Nest ID	4.457 ± 2.111	1199	51.00	36.140 ± 6.011	1377	46.29
Year	0.391 ± 0.625	1199	0.01	0.001 ± 0.001	1377	0.01
Residual	8.370 ± 2.893	1199	48.99	41.920 ± 6.475	1377	53.70

Abbreviations: NA, nestling age; *R*, average rainfall; *T*, average temperature; WS, average wind speed.

### 3.7 Female feeding preference under different nest sex ratios

The findings revealed that the female parental feeding was strongly negatively correlated with offspring type (*p* < .01), indicating that in female‐biased nests, females tended to feed marginal nestlings. There was a strong positive correlation with nestling sex (*p* < .01), showing a preference for feeding male nestlings.

In male‐biased nests, the feeding by female parents was influenced by the nest sex ratio, offspring type, and nestling age. The relationship with offspring type was strongly negative (*p* < .01), showing a preference for feeding marginal offspring. There was a strong positive correlation with both nest sex ratio and nestling age (*p* < .01). In nests with a male‐biased sex ratio, as nestling age increased, female feeding increased. However, in male‐biased nests, female feeding showed no correlation with nestling sex (Table 6).

**TABLE 6:** Factors affecting male parental investment preference under biased nest sex ratio.

Female‐biased nest sex ratio					Male‐biased nest sex ratio			
Fixed effects	*β* ± SE	*t*	*n*	*p*	*β* ± SE	*t*	*n*	*p*
Intercept	2.619 ± 0.127	20.569	262	<.001	2.413 ± 0.275	8.777	1408	.003
NA	0.003 ± 0.024	0.120	262	.905	0.034 ± 0.009	3.950	1408	<.001
Sex	0.325 ± 0.105	3.109	262	.002	−0.040 ± 0.052	−0.772	1408	.440
Sex ratio	−1.819 ± 0.621	−2.930	262	.071	0.760 ± 0.096	7.887	1408	<.001
NT	−2.408 ± 0.136	−17.675	262	<.001	−2.256 ± 0.044	−50.886	1408	<.001

Random effect	*β* ± SD	*n*	VCA (%)	*β* ± SD	*n*	VCA (%)
Nest ID	0.040 ± 0.200	262	51.00	0.002 ± 0.050	1408	0.37
Year	0.001 ± 0.001	262	0.01	0.224 ± 0.473	1408	33.18
Residual	0.651 ± 0.807	262	48.99	0.448 ± 0.669	1408	66.45

Abbreviations: NA, nestling age; NT, nestling type.

### 3.8 Male feeding preference under different nest sex ratios

In these nests, male feeding was strongly negatively correlated with both nest sex ratio and offspring type (*p* < .01) and strongly positively correlated with nestling sex (*p* < .01). Males displayed a preference for feeding male marginal offspring.

In male‐biased nests, male feeding was influenced by factors such as nest sex ratio, offspring type, nestling sex, average temperature, and nestling age. The relationship with offspring type and average temperature was strongly negative (*p* < .01), with males showing a preference for feeding marginal offspring. The feeding behavior showed a significant positive correlation with nest sex ratio, nestling age, and nestling sex (*p* < .05). As nestling age increased and in nests with a male‐biased ratio, males favored feeding male marginal offspring (Table 7).

**TABLE 7:** Factors affecting female parental investment preference under biased nest sex ratio.

Female‐biased nest sex ratio					Male‐biased nest sex ratio			*p*
Fixed effects	*β* ± SE	*t*	*n*	*p*	*β* ± SE	*t*	*n*	
Intercept	2.412 ± 0.059	40.976	199	<.001	2.507 ± 0.271	9.265	764	.003
NA	0.043 ± 0.037	1.140	199	.255	0.041 ± 0.011	3.656	764	<.001
Sex	0.674 ± 0.137	4.925	199	<.001	0.160 ± 0.067	2.374	764	.018
Sex ratio	−1.296 ± 0.308	−4.206	199	<.001	0.367 ± 0.100	3.656	764	<.001
NT	−2.319 ± 0.204	−11.388	199	<.001	−2.213 ± 0.057	−39.093	764	<.001
*T*	0.078 ± 0.069	1.133	199	.259	−0.094 ± 0.031	−3.065	764	.002

Random effect	*β* ± SD	*n*	VCA (%)	*β* ± SD	*n*	VCA (%)
Nest ID	0.001 ± 0.001	199	0.01	0.001 ± 0.001	764	0.01
Year	0.001 ± 0.001	199	0.01	0.218 ± 0.466	764	34.40
Residual	0.690 ± 0.830	199	99.98	0.415 ± 0.644	764	65.59

Abbreviations: NA, nestling age; NT, nestling type; *T*, average temperature.

## 4 DISCUSSION

Our egg data analysis indicates that there are differences in the number of eggs per nest and initial egg weight between 2021 and other years. Contrary to the large clutch‐small egg and small clutch‐large egg strategies observed in previous years, a trend from small clutch‐small egg towards large clutch‐large egg emerged (Table 2). This aligns with findings from Liu et al. (2005) in their study on the *Tragopan* genus (Precocial Birds), suggesting that the unique climatic environment of the Tibetan Plateau influences parental egg‐laying strategies. Under harsh environmental conditions, parents tend to lay larger clutches with heavier eggs, which may enhance nestling survival rates. Additionally, parents employ a descending order strategy in egg‐laying, where the initial eggs are heavier than the subsequent ones. Correspondingly, the nestlings born from earlier eggs also have a higher birth weight. Adopting this strategy can effectively reduce reproductive losses in the Gray‐backed Shrike (Du et al., 2014). Our results also show that average rainfall significantly affects clutch size: the lower the rainfall, the larger the clutch (Table 2). Reduced rainfall, to some extent, signals a decline in food availability, presenting adverse breeding conditions for the shrikes, a conclusion contrasting with findings in Sprague's Pipit (*Anthus spragueii*; Gaudet et al., 2020). The decision to lay larger clutches might be influenced by the parents' physical condition and possibly by their anticipation of better environmental conditions for nestling‐rearing in June. To enhance nestling survival, parents might increase their reproductive investment under optimal breeding conditions, thereby maximizing their fitness (Figure 1; Barkowska et al., 2003; Song et al., 2016).

Our study reveals that core offspring exhibit larger physical dimensions (weight, wing length, and beak gape) compared to marginal offspring (Figure 2). This suggests that under asynchronous hatching, core offspring hatch first and enjoy a developmental advantage. While there is a significant difference in nestling birth weight (*p* < .05), parents generally adopt a nest survival strategy. Marginal offspring receive a disproportionate share of resources from parents, resulting in faster growth rates in weight, wing length, and beak gape (Figure 3). Consequently, by the time of fledging, there is little difference in the weight of core and marginal offspring (Figure 2), and survival rates also show no significant disparities (Anderson et al., 1997; Nilsson & Svensson, 1996b; Stenning, 1996).

Male nestlings are larger than their female counterparts (Figure 2), and survival rates differ between sex. Specifically, male nestling survival rates exceed those of females by over 27% (Table 6). This might be attributed to a higher fitness return when rearing male nestlings under favorable environmental conditions (Dijkstra et al., 1998). The preferential feeding by male parents plays a role in this. By safeguarding the survival of male nestlings and increasing reproductive investment in them, male parents might be leveraging the fact that, within the Gray‐backed Shrike population, male nestlings can aid parents in occupying and expanding their reproductive territories (Bartlett et al., 2005). Additionally, promoting the dispersal of female nestlings might also serve as a strategy to prevent inbreeding (Gerlach & Hoeck, 2001).

Our study indicates a significant negative correlation between average temperature and nestling weight (*p* < .05; Table 3), suggesting that higher average temperatures hinder nestling growth and development. Stable environments are crucial for nestling maturation (Mertens, 1969). There are fewer shrike parents breeding in Luqu during July and August. This might be due to parents seeking to provide ideal developmental conditions for their nestlings, avoiding excessively high average temperatures and minimal average rainfall, and sourcing food that aligns with the nestling's growth needs (Naef‐Daenzer & Keller, 1999). Additionally, given Luqu's plateau environment, average temperatures drop rapidly in September. Nestlings soon face the challenge of molting in the winter, which is not conducive to their survival (Kiat et al., 2019). There is a highly significant positive correlation between nestling age and weight (*p* < .01; Table 3). As nestlings age, their metabolic rate accelerates, aligning with standard growth patterns and consistent with the feeding patterns of their parents.

Our research demonstrates a highly significant positive correlation between both male and female parental feeding amounts and the factors of nestling count, age, and sex ratio (*p* < .01; Table 5). As the number of nestlings increases, and as nestlings age, especially with a higher proportion of male nestlings, parents need to forage more food to ensure the growth and development of the nestlings. Male parental feeding amounts correlate significantly positively with average temperature (*p* < .01; Table 6). Higher average temperatures can boost plant growth, benefit insect development, and consequently increase the shrikes’ food sources, a finding that aligns with studies on the Red‐Backed Shrike (*Lanius collurio*; Hušek & Adamík, 2007). On the contrary, parental feeding amounts exhibit a significant negative correlation with wind speed (*p* < .05; Table 5). In high winds, the environment tends to be more challenging, and parents often shield their nestlings from the wind to prevent heat loss, which leads to reduced investment in other aspects, such as feeding rates decreasing (Heenan & Seymour, 2012; Saraux et al., 2016). Moreover, strong winds can hinder parental feeding (Heenan & Seymour, 2012; Saraux et al., 2016). Female parental feeding amounts negatively correlate with begging intensity (*p* < .05; Table 7), possibly related to parents regulating sibling competition among nestlings and reducing predator detection. This observation is consistent with studies on the Little Egret (*Egretta garzetta*; Mock & Lamey, 1991).

This study explores the feeding preferences of female parents. The feeding by female parents is influenced by the overall nest sex ratio. In nests with varying sex ratios, females tend to feed nestlings of the less represented sex, showing no clear preference for nestling sex. This finding aligns with research on the Black‐throated Tit (*Aegithalos concinnus*; Li et al., 2019). However, female parents exhibit a tendency to prioritize feeding the marginal offspring. In favorable environments, female parents adopt a nest survival strategy to enhance nestling survival rates (Nilsson, 1993). In nests with a higher proportion of male nestlings, the feeding by female parents is also influenced by the age of the nestlings. As nestlings age, female parents increase their investment, especially for male nestlings which are larger in size, have faster metabolisms, and require more food (Schmidt et al., 2012).

This study reveals that male parental feeding preferences are influenced by the type of offspring, prioritizing feeding marginal offspring and adopting a nest survival strategy consistent with female parents (Nilsson, 1993). Male parental feeding is also influenced by the nest sex ratio. Furthermore, male parental feeding preferences are affected by nestling sex. In nests skewed towards males or females, there is a pronounced preference for feeding male nestlings, which subsequently impacts the survival rates of different sex nestlings. The results suggest that in favorable environmental conditions, male parents prioritize nurturing male offspring to enhance their own fitness, a behavior consistent with findings in the Brown Falcon (*Falco berigora*; McDonald et al., 2005). Under higher average temperatures, male parents tend to favor feeding core offspring, possibly because larger nestlings in good conditions require more food (Schroeder et al., 2012). Also, as nestlings age, in male‐skewed nests, the frequency of feeding by male parents increases, aligning with the feeding tendencies of female parents (Schmidt et al., 2012).

## 5 CONCLUSION

In this study, we broadly divided the parental breeding phase into two periods. During the early breeding stage (incubation phase), parents adopt a nest reduction strategy due to suboptimal overall environmental conditions. They increase overall investments to ensure that a certain number of eggs successfully hatch, opting for larger clutch sizes with bigger eggs and a nest reduction laying strategy to minimize losses from initial investments. Once the nestlings hatch in the nestling‐rearing phase, conditions improve with more available food. Parents then enhance their contributions, using a nest survival strategy to promote the survival of the entire brood, ensuring the maximization of their own fitness. Additionally, male parents consistently exhibit a strong preference for feeding male nestlings in nests of any sex ratio (*p* < .01), while female parents don't show a clear nestling preference. The feeding preference of male parents aims to enhance their own fitness, leading to differences in survival rates among different sex nestlings under this preferential feeding.

## AUTHOR CONTRIBUTIONS

**Jinyuan Zeng:** Data curation (lead); formal analysis (lead); investigation (lead); methodology (lead); software (equal); writing – original draft (lead). **Yueqi Li:** Data curation (equal); investigation (equal). **Yuhan Gong:** Data curation (equal); investigation (equal). **Yurou Shi:** Data curation (equal); investigation (equal). **Hongyuan Feng:** Data curation (equal); investigation (equal). **Sen Song:** Data curation (equal); funding acquisition (lead); investigation (equal); resources (lead); supervision (lead); writing – review and editing (lead).

## FUNDING INFORMATION

The National Natural Science Foundation of China (Grant 31301889), The Natural Science Foundation of Gansu Province (22jr5ra468), Gansu Ziwuling Ecosystem Observation and Research Station (20JR10RA658).

## CONFLICT OF INTEREST STATEMENT

The authors declare no conflict of interest.

## Supporting information


Data S1.

Table S1.

Table S2.

Table S3.

## Data Availability

All data in this study are included as supplementary information files.
