# Supplementary material for: Variation in parental investment preferences for nestlings of the Gray‐backed Shrike (Lanius tephronotus) in alpine environments
Source: Ecol Evol. 2024 Sep 18;14(9):e70267. doi: 10.1002/ece3.70267 (PMC11410560; doi:10.1002/ece3.70267)
Supplement: Supplementary file 2 — Table S1. [file ECE3-14-e70267-s003.docx]

| Component | Status | Score |
| --- | --- | --- |
| Mouth: | Open/Not | (1/0) |
| Head: | Raised/Not | (1/0) |
| Neck: | Extended/Not | (1/0) |
| Body: | Upright/Not | (1/0) |
| Legs: | Upright/Not | (1/0) |
| Tarsi: | Upright/Not | (1/0) |


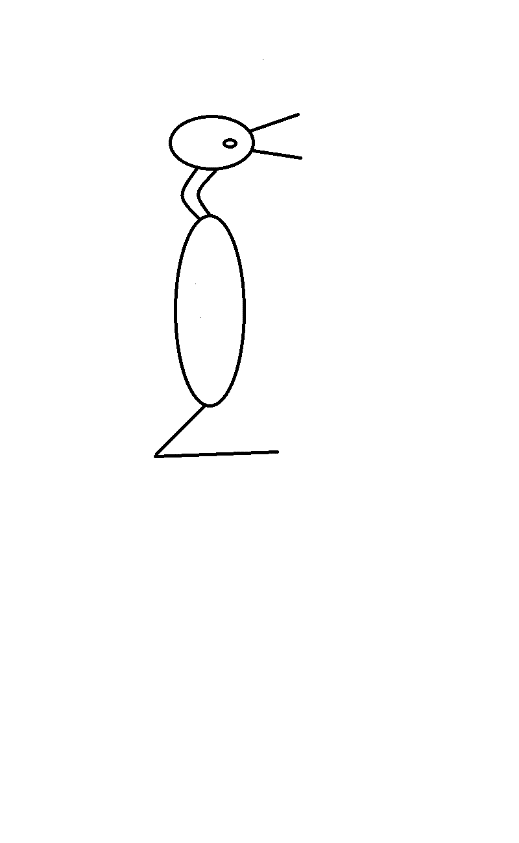


Table S1 Scores of Nestling Begging Intensity
