# Supplementary material for: Variation in parental investment preferences for nestlings of the Gray‐backed Shrike (Lanius tephronotus) in alpine environments
Source: Ecol Evol. 2024 Sep 18;14(9):e70267. doi: 10.1002/ece3.70267 (PMC11410560; doi:10.1002/ece3.70267)
Supplement: Supplementary file 3 — Table S2. [file ECE3-14-e70267-s004.docx]

| Dependent Variable | Hatching Order | | Mean Difference (I-J) | Standard Error | Significance | 95% Confidence Interval | |
| --- | --- | --- | --- | --- | --- | --- | --- |
|  |  |  |  |  |  | Lower Bound | Upper Bound |
| wing length | 1 | 2 | -1.0464 | 1.60143 | 0.514 | -4.1906 | 2.0979 |
|  |  | 3 | 0.9336 | 1.65341 | 0.572 | -2.3127 | 4.1799 |
|  |  | 4 | 2.4134 | 1.80507 | 0.182 | -1.1307 | 5.9575 |
|  |  | 5 | 0.6009 | 1.93572 | 0.756 | -3.1997 | 4.4015 |
|  | 2 | 1 | 1.0464 | 1.60143 | 0.514 | -2.0979 | 4.1906 |
|  |  | 3 | 1.9799 | 1.64065 | 0.228 | -1.2413 | 5.2012 |
|  |  | 4 | 3.4598 | 1.79339 | 0.054 | -0.0614 | 6.9809 |
|  |  | 5 | 1.6473 | 1.92483 | 0.392 | -2.1319 | 5.4265 |
|  | 3 | 1 | -0.9336 | 1.65341 | 0.572 | -4.1799 | 2.3127 |
|  |  | 2 | -1.9799 | 1.64065 | 0.228 | -5.2012 | 1.2413 |
|  |  | 4 | 1.4798 | 1.83996 | 0.422 | -2.1327 | 5.0924 |
|  |  | 5 | -0.3327 | 1.96829 | 0.866 | -4.1972 | 3.5319 |
|  | 4 | 1 | -2.4134 | 1.80507 | 0.182 | -5.9575 | 1.1307 |
|  |  | 2 | -3.4598 | 1.79339 | 0.054 | -6.9809 | 0.0614 |
|  |  | 3 | -1.4798 | 1.83996 | 0.422 | -5.0924 | 2.1327 |
|  |  | 5 | -1.8125 | 2.09730 | 0.388 | -5.9303 | 2.3053 |
|  | 5 | 1 | -0.6009 | 1.93572 | 0.756 | -4.4015 | 3.1997 |
|  |  | 2 | -1.6473 | 1.92483 | 0.392 | -5.4265 | 2.1319 |
|  |  | 3 | 0.3327 | 1.96829 | 0.866 | -3.5319 | 4.1972 |
|  |  | 4 | 1.8125 | 2.09730 | 0.388 | -2.3053 | 5.9303 |
| beak gape | 1 | 2 | -0.1077 | 0.39159 | 0.783 | -0.8765 | 0.6612 |
|  |  | 3 | 0.1934 | 0.40430 | 0.632 | -0.6004 | 0.9872 |
|  |  | 4 | 0.6448 | 0.44139 | 0.144 | -0.2218 | 1.5114 |
|  |  | 5 | 0.6812 | 0.47333 | 0.151 | -0.2481 | 1.6105 |
|  | 2 | 1 | 0.1077 | 0.39159 | 0.783 | -0.6612 | 0.8765 |
|  |  | 3 | 0.3011 | 0.40118 | 0.453 | -0.4866 | 1.0888 |
|  |  | 4 | 0.7525 | 0.43853 | 0.087 | -0.1085 | 1.6135 |
|  |  | 5 | 0.7889 | 0.47067 | 0.094 | -0.1352 | 1.7130 |
|  | 3 | 1 | -0.1934 | 0.40430 | 0.632 | -0.9872 | 0.6004 |
|  |  | 2 | -0.3011 | 0.40118 | 0.453 | -1.0888 | 0.4866 |
|  |  | 4 | 0.4514 | 0.44992 | 0.316 | -0.4320 | 1.3348 |
|  |  | 5 | 0.4878 | 0.48130 | 0.311 | -0.4572 | 1.4327 |
|  | 4 | 1 | -0.6448 | 0.44139 | 0.144 | -1.5114 | 0.2218 |
|  |  | 2 | -0.7525 | 0.43853 | 0.087 | -1.6135 | 0.1085 |
|  |  | 3 | -0.4514 | 0.44992 | 0.316 | -1.3348 | 0.4320 |
|  |  | 5 | 0.0364 | 0.51284 | 0.943 | -0.9705 | 1.0433 |
|  | 5 | 1 | -0.6812 | 0.47333 | 0.151 | -1.6105 | 0.2481 |
|  |  | 2 | -0.7889 | 0.47067 | 0.094 | -1.7130 | 0.1352 |
|  |  | 3 | -0.4878 | 0.48130 | 0.311 | -1.4327 | 0.4572 |
|  |  | 4 | -0.0364 | 0.51284 | 0.943 | -1.0433 | 0.9705 |
| Dependent Variable | Brood size | | Mean Difference (I-J) | Standard Error | Significance | 95% Confidence Interval | |
|  |  |  |  |  |  | Lower Bound | Upper Bound |
| wing length | 2.00 | 3.00 | 5.9755 | 3.51475 | 0.090 | -0.9253 | 12.8763 |
|  |  | 4.00 | 3.6059 | 3.26693 | 0.270 | -2.8084 | 10.0202 |
|  |  | 5.00 | 4.4144 | 3.14616 | 0.161 | -1.7627 | 10.5916 |
|  | 3.00 | 2.00 | -5.9755 | 3.51475 | 0.090 | -12.8763 | 0.9253 |
|  |  | 4.00 | -2.3696 | 2.06363 | 0.251 | -6.4214 | 1.6821 |
|  |  | 5.00 | -1.5611 | 1.86656 | 0.403 | -5.2259 | 2.1037 |
|  | 4.00 | 2.00 | -3.6059 | 3.26693 | 0.270 | -10.0202 | 2.8084 |
|  |  | 3.00 | 2.3696 | 2.06363 | 0.251 | -1.6821 | 6.4214 |
|  |  | 5.00 | 0.8086 | 1.34293 | 0.547 | -1.8281 | 3.4453 |
|  | 5.00 | 2.00 | -4.4144 | 3.14616 | 0.161 | -10.5916 | 1.7627 |
|  |  | 3.00 | 1.5611 | 1.86656 | 0.403 | -2.1037 | 5.2259 |
|  |  | 4.00 | -0.8086 | 1.34293 | 0.547 | -3.4453 | 1.8281 |
| beak gape | 2.00 | 3.00 | 0.6941 | 0.85944 | 0.420 | -0.9933 | 2.3815 |
|  |  | 4.00 | -0.1648 | 0.79885 | 0.837 | -1.7333 | 1.4036 |
|  |  | 5.00 | 0.4542 | 0.76932 | 0.555 | -1.0563 | 1.9647 |
|  | 3.00 | 2.00 | -0.6941 | 0.85944 | 0.420 | -2.3815 | 0.9933 |
|  |  | 4.00 | -0.8589 | 0.50461 | 0.089 | -1.8497 | 0.1318 |
|  |  | 5.00 | -0.2399 | 0.45642 | 0.599 | -1.1360 | 0.6563 |
|  | 4.00 | 2.00 | 0.1648 | 0.79885 | 0.837 | -1.4036 | 1.7333 |
|  |  | 3.00 | 0.8589 | 0.50461 | 0.089 | -0.1318 | 1.8497 |
|  |  | 5.00 | 0.6191 | 0.32838 | 0.060 | -0.0257 | 1.2638 |
|  | 5.00 | 2.00 | -0.4542 | 0.76932 | 0.555 | -1.9647 | 1.0563 |
|  |  | 3.00 | 0.2399 | 0.45642 | 0.599 | -0.6563 | 1.1360 |
|  |  | 4.00 | -0.6191 | 0.32838 | 0.060 | -1.2638 | 0.0257 |

Table S2: Results of the Multivariate Analysis of Variance (MANOVA) for Wing Length and Beak Gape.
