# Supplementary material for: Variation in parental investment preferences for nestlings of the Gray‐backed Shrike (Lanius tephronotus) in alpine environments
Source: Ecol Evol. 2024 Sep 18;14(9):e70267. doi: 10.1002/ece3.70267 (PMC11410560; doi:10.1002/ece3.70267)
Supplement: Supplementary file 4 — Table S3. [file ECE3-14-e70267-s002.docx]

**Table S3.** The mean mass of each food type delivered to the brood by the Grey-backed Shrikes.

| **Types of food** | **Mean mass weighed** |
| --- | --- |
| Larvae | 0.1 g/two individuals |
| Large Larvae/Small Adults | 0.8 g/per individual |
| Medium Adults | 1.22 g/per individual |
| Large Adults | 2.25 g/per individual |
